# A Latent Profile Analysis of Anxiety among Junior High School Students in Less Developed Rural Regions of China

**DOI:** 10.3390/ijerph17114079

**Published:** 2020-06-08

**Authors:** Xiaotong Wen, Yixiang Lin, Yuchen Liu, Katie Starcevich, Fang Yuan, Xiuzhu Wang, Xiaoxu Xie, Zhaokang Yuan

**Affiliations:** 1 School of Public Health, Jiangxi Province Key Laboratory of Preventive Medicine, Nanchang University, Nanchang 330006, China; 406530517824@email.ncu.edu.cn (X.W.); 401440318202@email.ncu.edu.cn (Y.L.); 2 Biology Department, Mellon College of Science, Carnegie Mellon University, Pittsburgh, PA 15213, USA; renata2001bella@hotmail.com; 3 School of Community Science, University of Nevada, Reno, NV 89557, USA; Kstarcevich@nevada.unr.edu; 4 Office of Public Health Studies, the University of Hawaii at Mānoa, Honolulu, HI 96822, USA; fangy@hawaii.edu; 5 Administration Office of Floating Population, Jiangxi Provincial Health Committee, Nanchang 330006, China; jsw_lgc@jiangxi.gov.cn; 6 School of Public Health, Fujian Medical University, Fuzhou 350000, China

**Keywords:** rural, junior high school students, anxiety, latent profile analysis, academic pressure

## Abstract

The purpose of this study is to understand the potential types of anxiety among middle school students by analyzing the current situation of middle school students’ anxiety and its influencing factor. This study used a multistage stratified cluster random sampling to investigate students in grades 9 to 12. Mplus 7.4 was used for latent profile analysis. A total of 900 junior high school students were investigated. The junior high school students were divided into three subgroups by latent profile analysis. A total of 223 junior high school students experienced severe anxiety, accounting for 24.78%. Multivariate logistic regression analysis revealed that males are more likely to develop moderate and severe anxiety. The development of severe anxiety (OR = 0.562, *p* < 0.05) is less likely for students in schools with adequate mental health support. Students who were confident with their academic performances were less likely to develop moderate anxiety (OR = 0.377, *p* < 0.05). Students with extreme academic pressure are more likely to develop moderate anxiety (OR = 6.523, *p* < 0.05) and severe anxiety (OR = 11.579, *p* < 0.05). It is recommended that mental health counseling be set up in schools and to provide professional counselors to prevent serious anxiety for students. This paper also demonstrates a need to reduce students’ academic pressure.

## 1. Introduction

Junior high school is a critical time for individual growth and development, and also an important stage for the formation of individual personality and values. However, families and schools focus mainly on the physical growth, academic performance, and health-related behavior of junior high students. The mental and emotional health of the students is seriously ignored in comparison, resulting in them facing psychological confusion and mental health problems. Many of the students bear the burden alone and fail to cope with it properly, which leads to more serious anxiety [1]. Although the concept of general education has been continuously promoted, the academic pressure for students continues to increase. Families tend to put high expectations on their children, who face stress in schools as well as in society. The Chinese education system is widely criticized by its own educators, scholars, and parents for focusing on academics over other pursuits, generating toxic levels of stress, disregarding students’ emotional wellbeing, which may produce graduates with high scores and poor health [2]. One potential result is the high incidence of anxiety among students [3]. Junior high school students were observed to be at high risk for anxiety, which be attributed, at least in part, to the nature of the Chinese school system [4]. Therefore, students’ emotional and psychological problems are getting worse, especially their anxiety issues, which need to be taken seriously by families, schools, and society. 

Anxiety refers to a kind of irritability that is exhibited through fear, uneasiness, and excessive worry about things that will have negative consequences or vague threats [5,6]. Mild anxiety can be a driving force to adapt to the environment, but severe anxiety can seriously affect physical and mental health [7]. Studies have shown that anxiety is a good predictor of suicidal tendencies [8]. Currently, anxiety is common throughout the world, and is the most common mental health problem. A systematic review of epidemiological studies in 44 countries found that the global prevalence of severe anxiety is estimated at 7.3%, which indicates that one in every 14 people in the world suffers from severe anxiety [9]. The prevalence of severe anxiety in the United States reached 31.0%, and in Europe, 14.0% [10,11]. A cross-sectional survey in Tanzania found that the anxiety rate of 3013 adolescents was 31.0% [12]. Currently, the anxiety of adolescents is becoming a serious public health concern, and further attention from the public is needed. 

Understanding the influencing factors of anxiety among junior high school students can guide them to actively prevent and cope with anxiety. The factors that affect anxiety may come from personal issues, family, school, etc. Yao Shuqiao [13] investigated the symptoms of anxiety in Chinese teenagers and found that girls had more anxiety symptoms than boys. Analysis by Petermann also found that females are at higher risk for anxiety [14]. Recent research by Cheung found that lifestyle is significantly associated with anxiety, with the results suggesting that individuals who sleep less than 7–8 h have fewer hobbies, rarely spend time engaged in entertainment, and are more likely to develop anxiety [15]. In addition, a randomized control study of individuals with anxiety and depression that applied lifestyle changes has shown that a healthy diet and physical exercise can reduce anxiety levels in patients [16,17]. A follow-up study by Tochigi found that sleeping habits are significantly associated with adolescent anxiety, showing that delays in bedtime aggravated anxiety for middle school students, and pointed out that improving adolescents’ sleep habits has an important role in improving their mental health [18]. Some studies have also found that incomplete family structure is a risk predictor of anxiety. The results show that females with a single parent or without parents are more likely to develop anxiety [19]. Current research indicates that personal factors that are related to the anxiety of adolescents include gender, age, lifestyle, and sleep status, and family factors include family structure, environment, and socioeconomic status. This article will mainly explore the effects of school-related factors on the anxiety level of junior high school students. Evidence suggests that high levels of anxiety are unusually prevalent among students in China. The survey which investigated 1576 junior high school students in China found that a high portion of students has severe anxiety symptoms, such as sleep difficulties (accounting for 27%) and emotional questions (accounting for 48%) [20]. Previous studies have shown that anxiety rates are higher among rural students than urban students [20,21]. Many students in junior high school may begin to realize the anxiety in their future career and life. The junior high school students need to pass a cut-off score to enter academically advanced high schools. 

The issue of anxiety in Chinese teenagers is escalating. Most domestic studies on anxiety have focused on urban areas, and there is a lack of attention to anxiety in rural middle school students. Adolescents in rural areas require more attention. This project conducts a survey on junior high school students in Jiangxi rural areas to understand the current status of anxiety issues among them. It also explores how moderate/severe anxiety is associated with basic demographic information and school-related factors. Exploratory latent profile analysis is proposed to further investigate the characteristics of the anxiety of rural junior high school students in Jiangxi Province. By using latent continuous variables to explain the relationship between explicit continuous variables, it can make full use of all the sample data and make estimates of what certain group an individual belongs in and, thus, explore heterogeneous classifications within groups. Based on the result data, we aim to find the potential classification of anxiety, describe the overall level of anxiety among rural students, explore the influencing factors of anxiety among different groups, and thus provide a basis and measures for effective intervention.

Accordingly, we had four hypotheses. First, we hypothesized that in the cross-sectional study, the number of potential classifications of anxiety among junior high school students would be three. Secondly, we hypothesized that students would be a low level of anxiety when school mental health work is adequate. Thirdly, we hypothesized that students with excellent academic performance would be more anxious than students with failure academic performance. Finally, we hypothesized that students with high academic pressure would be more anxious.

## 2. Materials and Methods

### 2.1. Research Targets and Sampling Methods

**Figure 1:**
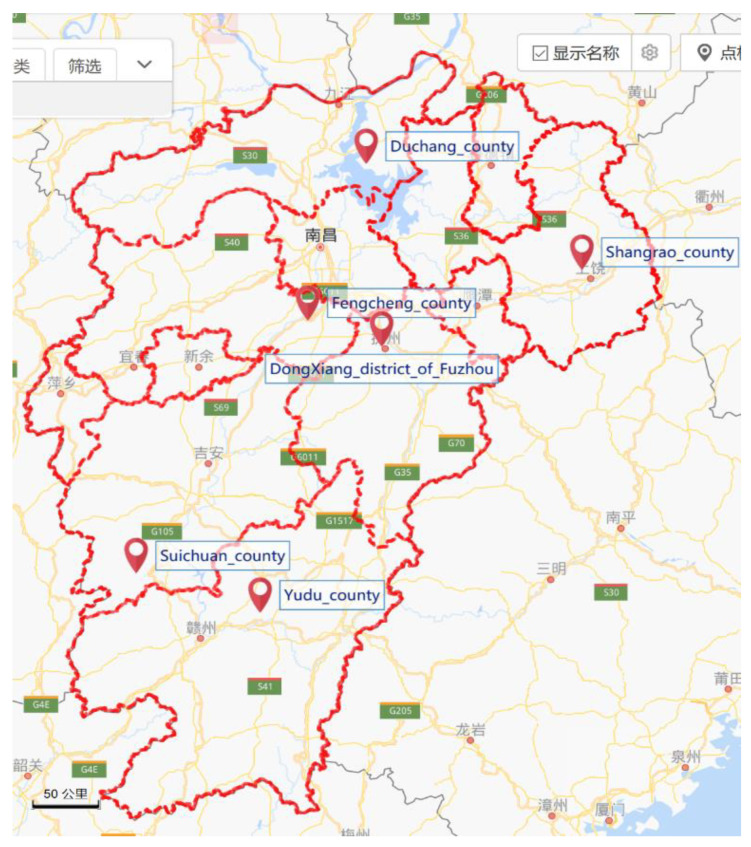
Distribution of sample counties in Jiangxi Province.

The survey targets students in grades 9 to 12 in a total of six counties: Yudu, Shangrao, Duchang, Fengcheng, Dongxiang District in Fuzhou, and Suichuan (Figure 1). In this survey, the Jiangxi Provincial Health Committee’s Administration office of the floating population determined the sample counties for the survey, and the sample counties then determine a sample township. A middle school in the town was selected as the sample school, and sample classes in each grade were determined by simple random sampling.

A simple random sampling method was used to determine the sample class in each grade of the sample school. First, the number of students in every class in the sample school is collected, and the total number of students in each grade is calculated. If there are hundreds of students in this grade, a three-digit random number will be selected. If there are dozens of students in this grade, a two-digit random number will be selected. Second, we selected a row and column at random in random number table and determined a random number. Finally, students are added one by one until the number of students in the class of this grade becomes larger than the random number; the class selection in every grade has then been determined. All the students in this study were recruited in randomly selected classes. 

There were 900 junior high school students in rural areas of Jiangxi Province selected in this survey. The average age of the sample students was 14.14 ± 1.32 years old.

### 2.2. Research Methods

The survey adopted a multistage stratified cluster random sampling method and conducted a questionnaire survey by conducting field surveys in schools. The local Health Committee, Center for Disease Control and Prevention, Ministry of Education, and other departments jointly assisted in conducting the field investigation. The investigator, with the assistance of the class teachers, organized the students in class to participate in the research. The investigator showed the correct way of answering questions on the blackboard for the students. The students were informed that the questionnaires were anonymous, that the results of the questionnaire would not be used as a basis for judging their academic performance, and that there was no correct answer to the questions. They were asked to complete the survey truthfully and independently, without discussion or interaction. The investigator checked the completeness of the questionnaires immediately after students finished. If there were any missing questions, students were asked to complete them to ensure the integrity of the survey. The junior high school students were told that participation was anonymous and voluntary. The parents had provided consent at the midterm parents meeting. In other words, parental consent to participate in the survey was obtained in advance. Ethical approval of this study was approved by the Nanchang University Institutional Review Board. 

### 2.3. Content of Questionnaires

The questionnaire includes two parts: the first part is the basic demographic characteristics (including gender, age, grade, left-behind children) and school-related factors of rural middle school students; the second part is the Mental Health Test (MHT) [22,23]. According to the Diagnostic Test of Anxiety Tendency which was compiled by Japanese researcher Kiyoshi Suzuki, Professor Bucheng Zhou and other psychology researchers of the Department of Psychology of East China Normal University adapted the questionnaires to establish the Chinese version in the year of 1991, and this questionnaire was named the Mental Health Test, which is a standardized anxiety diagnostic scale for primary and middle school students in China. The MHT is an internationally standardized test for the anxiety of children that has been widely applied in China [24]. The MHT has a total of 100 questions, consisting of 8 content scales and 1 effectiveness scale. The MHT measures anxiety from two aspects: anxiety objects and anxiety behaviors. Anxiety objects are learning anxiety and interpersonal anxiety. Anxiety behaviors include lonely tendency, remorse tendency, allergic tendency, physical symptoms, terror tendency, and impulsive tendency. 

According to the MHT scale, learning anxiety refers to a student’s fear of examinations or excessive concerns about test scores. This includes being worried about passing exams successfully, feeling unhappy when the test scores are not good, a student feeling anxious when they cannot remember what they have learned during an examination, and worrying about getting a poor score when taking an exam. Interpersonal anxiety refers to a student having difficulties in communicating with others, such as fear of strangers, blushing when talking to others, always having teachers’ or parents’ blame on the mind. Lonely tendency refers to a student feeling that it is better to play alone than with others, fearing failure when joining in the team. Remorse tendency refers to students losing confidence in what they do, often worrying about this which hinders their actions. Allergic tendency refers to students that are too sensitive, such as particularly sensitive to the noise around them, worrying that some of their family members injured, sick, or dead. Physical symptoms refer to a student’s excessive concerns about his/her body, such as always being worried about whether there is something wrong in his/her body, having difficulty breathing, sweating, dizziness and abnormal perception, restlessness, abnormal heartbeat, disordered pulse, vomiting, anorexia, stomachache or insomnia. Terror tendency refers to students who are always in a state of fear without any reasons. Impulsive tendency refers to a student’s desire to do dangerous or stupid things without any reason, which is due to the internal anxiety tendency.

The MHT is a self-assessment scale which is easy to operate and easy for subjects to accept and master. Each entry has two points, (Yes = 1 and No = 0). This test has good reliability and validity indicators. The test has a reliability of 0.84–0.88 and retest reliability of 0.78–0.86 [24]. After confirmatory factor analysis, the overall fitting index of the scale is as follows: *χ^2^* = 19239.961, df = 4950, CFI = 0.913, GFI = 0.931, NFI = 0.905, RMSEA = 0.048, and the internal consistency reliability as indicated by Cronbach’s α coefficient is 0.878. Confirmatory factor analysis shows that the reliability coefficients of the eight content scales are learning anxiety (0.847), interpersonal anxiety (0.757), lonely tendency (0.789), remorse tendency (0.777), allergic tendency (0.770), physical symptoms (0.794), terror tendency (0.840), and impulsive tendency (0.839).

### 2.4. Index Definition

#### 2.4.1. Left-Behind Children (LBC)

Left-behind children are defined as those children under 16 years of age who are left at home when both parents migrate to an urban area for work for more than 6 months per year, or when one of them migrates to an urban area for work for more than 6 months per year and the other does not have the ability to bring up and supervise the children [25,26].

#### 2.4.2. School-Related Factors

The health education course was assessed by asking students: “Does your school offer a health education course?” The answers for this question were divided into “Yes” and “No”. Whether school mental health work is adequate or not was assessed by asking students: “Do you think school mental health work is adequate or not?” The two questions refer to the China Centers for Disease Control and Prevention adolescent mental health project. The answers for this question were divided into “Yes”, “No”, and “Not sure”. Self-assessment of academic performance of junior high school students was divided into four levels, “Excellent”, “Good”, “Pass”, and “Fail”. Self-assessment of the academic pressure of junior high school students was divided into five levels, “Very high”, “High”, “General”, “Low”, and “Very low”. The two questions refer to the US Centers for Disease Control and Prevention Youth Risk Behavior Surveillance System (YRBSS) questionnaire.

### 2.5. Data Analytic Approach

Epidata 3.1 (The EpiData Association, Odense, Denmark) was employed to input data. The database was imported into Excel spreadsheets (Microsoft Office 2003, Microsoft, Redmond, DC, USA) and transferred to SPSS 24.0 statistical software (IBM Corporation, Armonk, NY, USA) for basic analysis. The latent profile approach (LPA) was conducted in Mplus 7.4 (Linda Muthén & Bengt Muthén) in order to explore the potential classification of anxiety among junior high school students. The differences between variables were compared using the *χ^2^* statistic method. Logistic regression was used in multifactor analysis with the test standard set as α = 0.05.

In recent years, there has been an increased use of person-centered statistical techniques in the field of psychology, including latent class analysis (LCA) and latent profile analysis (LPA) [27]. LCA and LPA, instead of focusing on correlations between study variables, identify typologies of individuals by examining configurations of traits within those individuals [28]. 

LPA is a “top-down” approach which requires the investigator to specify the number of hypothesized profiles in the data. For each participant, the probability of being in a given profile is estimated, and classification into one of the profiles is determined by that individual’s highest-profile probability [29]. LPA was used to detect homogeneous groups (latent classes) using eight factors of the anxiety, including learning anxiety, interpersonal anxiety, lonely tendency, remorse tendency, allergic tendency, physical symptoms, terror tendency, and impulsive tendency. By using a multinomial logistic regression, we assessed the association between latent classes of anxiety and school-related factors. These associations were evaluated using odds ratios (ORs), along with accompanying confidence intervals (CIs).

Several information indicators were used to evaluate the fit of latent profile models: Akaike information criterion (AIC), Bayesian information criterion (BIC), and same-size adjusted Bayesian information criterion (ABIC) [30,31,32]. The model with the lower value indicates the better latent profile solution. We tend to choose simple and effective models when we use AIC as the model adaptation standard. BIC is suitable for studies with a sample size of more than 1000 or with few model parameters, while ABIC requires at least 50 subjects in each category to ensure the accuracy of the evaluation model [33]. We also calculated the entropy value which indicates the accuracy of models, with a higher value indicating more accurate classification. Entropy values range from 0 to 1. When the entropy value is equal to 0.8, the classification accuracy of the model is more than 90%. Lo–Mendell–Rubin likelihood ratio test (LMRT), Vuong–Lo–Mendell–Rubin (VLMR) likelihood ratio test, and bootstrapped likelihood ratio test (BLRT) were used to compare models with increasing numbers of latent classes [34,35,36]. A significant value (*p* < 0.05) suggests that the model with one more class is a better choice. We preferred BIC as the best choice among information indicators. We preferred BLRT as the best choice when we compared models with different numbers of latent classes [37]. Furthermore, the most parsimonious model should be selected, and the smallest class of any class-solution should not contain less than 5% of the sample [38].

After the number and nature of the profiles were identified, individuals were assigned to their most likely profile based on their posterior probabilities (that is, the set of values describing the likelihood of being assigned to that profile, given the data) [39].

In this study, survey data were collected from questionnaires that students completed in class. The data were analyzed to detect anxiety homogeneous groups among junior high school students by latent class analysis and explore the association between latent classes of anxiety and school-related factors.

## 3. Results

### 3.1. Latent Profile Analysis (LPA) in the Anxiety of Junior High School Students in Rural Areas of Jiangxi Province

LPA was conducted on the entire sample. Table 1 reports commonly used fit statistics for 1 through 5 class solutions for analytic samples. As shown in Table 1, the lower value of the model information indicators, including AIC, BIC, and ABIC, the better the latent profile solution with increasing numbers of latent classes. In addition, the smallest class of every model does not contain samples of less than 5%. The entropy value is higher when the model changes from two to three latent classes. In other words, the entropy value confirms the supremacy of the three-class solution over alternative solutions. There were significant values (*p* < 0.001) for LMRT, VLMR, and BLRT, which suggests that the model with three latent classes was the better choice. On the basis of these statistics, the three-class solution is considered the best-fitting model.

**Figure 2:**
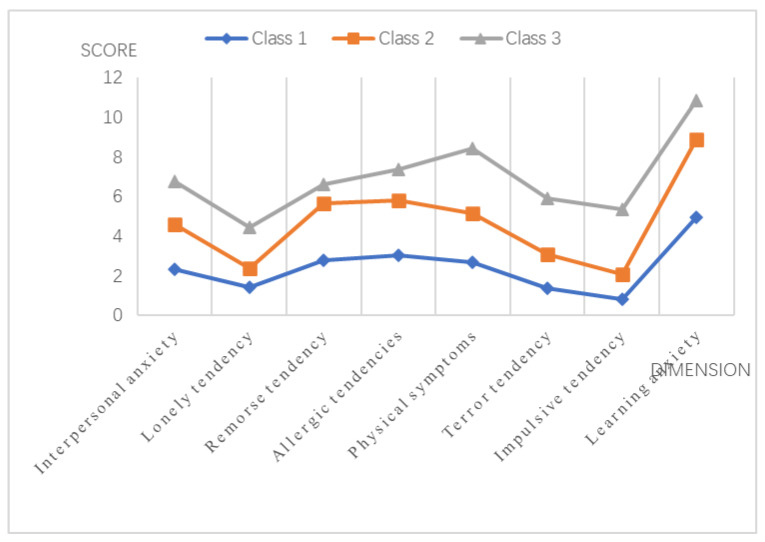
Depiction of the three latent classes defined by means on eight facets of anxiety among junior high school students in rural areas of Jiangxi Province.

The junior high school students were divided into three subgroups by latent profile analysis. Class 1 is the smallest group, which has 173 junior high school students, accounting for 19.22%. It is characterized by the lowest mean scores on all eight factors of the anxiety and is labeled the “mild anxiety” group. The eight factors of anxiety included learning anxiety, interpersonal anxiety, lonely tendency, remorse tendency, allergic tendency, physical symptoms, terror tendency, and impulsive tendency. Class 2 is the largest group, which has 504 junior high school students, accounting for 56.00%. It is characterized by moderate scores on all eight factors of the anxiety. This class is labeled the “moderate anxiety” group. Class 3 has 223 junior high school students, accounting for 24.78%. Class 3 is characterized by the highest mean scores on all eight factors of the anxiety. This class is labeled the “severe anxiety” group. Figure 2 shows the profile plot for the three-class solution.

### 3.2. The Univariate Analysis of Anxiety of Junior High School Students in Rural Area of Jiangxi Province

There were 900 junior high school students in rural areas of Jiangxi Province selected in this survey. The latent classes of the anxiety of junior high school students by latent profile analysis revealed that 173 students were mildly anxious, accounting for 19.22%, 504 students were moderately anxious, accounting for 56.00%, and 223 junior high school students were of severe anxiety type, accounting for 24.78%. 

The male to female ratio of 900 junior high school students was 1.08 to 1. The group aged 14 accounted for the highest proportion with 28.67%, and the group aged 12 had the lowest proportion with 14.00%. Among these junior high school students, 29.22% were left-behind children, 15.67% self-assessed academic pressure as very high. The students who thought that school mental health work was adequate accounted for 43.00%. The students who answered that school had a health education course accounted for 43.00%. The group whose self-assessment of academic performance was good accounted for the highest proportion, with 44.22%, and the group whose self-assessment of academic performance was excellent had the lowest proportion, with 4.56%.

As shown in Table 2, we identified the statistical difference in the classification of anxiety among students with the different gender group (*χ^2^* = 31.337, *p* < 0.001), the group who gave different answers to “school mental health work is adequate” (*p* < 0.001), the group with different self-assessment of their academic performance (*p* < 0.001), and academic pressure (*p* < 0.001).

### 3.3. The Multifactor Analysis of Anxiety of Junior High School Students in Rural Area of Jiangxi Province 

This analysis was conducted by taking anxiety as dependent variables and seven other factors as independent variables. The independent variable considers factors like gender, age, left-behind children, health education course, school mental health work is adequate, self-assessment of academic performance, self-assessment of academic pressure. It is preferable to analyze after transforming the categorical variable into dummy variables. The variables assignment summary for logistic regression analysis is shown in Table 3.

The independent variables were input into the equation for analysis, and the odds ratio (OR) of each independent variable was calculated. The logistics regression analysis of anxiety of junior high school students in rural areas of Jiangxi Province revealed that the probability of moderate anxiety in males is lower (OR = 0.649, *p <* 0.05) than for females, and the risk of severe anxiety in males is lower (OR = 0.262, *p <* 0.05) than for females. The logistics regression analysis of anxiety revealed that the probability of severe anxiety of junior high school students is lower (OR = 0.562, *p <* 0.05) if school mental health work is adequate. The logistics regression analysis of anxiety revealed that the probability of moderate anxiety of junior high school students whose self-assessment of academic performance is excellent was lower (OR = 0.377, *p <* 0.05). The junior high school students whose self-assessment of academic pressure is very high are more likely to have moderate anxiety (OR = 6.523, *p <* 0.05) and severe anxiety (OR = 11.579, *p <* 0.05). The junior high school students whose self-assessment of academic pressure is high are more likely to have moderate anxiety (OR = 6.122, *p <* 0.05) and severe anxiety (OR = 5.894, *p <* 0.05). Table 4 shows the results of the logistics regression analysis.

## 4. Discussion

In this study, we used latent profile analysis (LPA) to detect latent classes of anxiety among junior high school students in rural Jiangxi Province. This classification method was objective which used eight factors of the anxiety, including learning anxiety, interpersonal anxiety, lonely tendency, remorse tendency, allergic tendency, physical symptoms, terror tendency, and impulsive tendency. First, LPA yielded a three-class solution as the best fit to the data. A total of 173 junior high school students experienced mild anxiety, accounting for 19.22%; 504 junior high school students experienced moderate anxiety, accounting for 56.00%; 223 junior high school students experienced severe anxiety, accounting for 24.78%. A recent report has indicated that severe anxiety accounts for 35% of junior high school students, which is higher than the results of this survey [40].

A study of 1012 adolescents revealed that the rate of severe anxiety was higher for females than for males [41]. A previous study revealed that gender differences in anxiety rates, finding that girls were more anxious than boys [42]. The logistic regression analysis of this study revealed that the risk of moderate anxiety in males is lower than females, and the probability of severe anxiety in males is lower. Some findings support the importance of gonadal hormone involvement in guiding the brain within key stress and emotion regulatory regions, resulting in males doing better under stress and regarding regulation of emotions [43]. A study showed that the rates of severe anxiety among females were higher than males, which is consistent with the results of this study [44]. This may be due to females being more sensitive to their surroundings and events. Females are richer in emotional experience and more sensitive to emotional fluctuations. 

Some surveys have shown that school-related factors play a significant role in adolescent mental health [45,46,47]. Severe anxiety may disturb the study and life of adolescents. However, adolescents often do not receive counseling services when they have psychological problems (e.g., severe anxiety) [39]. This study revealed that junior high school students were at lower risk for severe anxiety when school mental health work was adequate. Schools should strengthen communication and carry out psychological counseling and psychotherapy [48]. The school develops mental health programs which can improve students’ positive psychological state and prevent severe anxiety [46,47]. In order to prevent students from severe anxiety, a psychological consultation room should be set up at the school. At the same time, psychological consultation should be with professional psychological counselors. A study in America revealed that students poor at academic performance are at higher risk at severe anxiety [49]. The probability of severe anxiety in students whose academic performance was good was 0.362 times than students whose academic performance failed. 

There is a key link between stress and severe anxiety [50]. A cross-sectional study showed that adolescents with academic pressure were at 2.4 times higher risk of severe anxiety than adolescents without academic pressure [51]. The students whose self-assessment of academic pressure was very high were 6.523 times more likely to have moderate anxiety than the students whose self-assessment of academic pressure was very low. The students whose self-assessment of academic pressure was very high were 11.579 times more likely to suffer from severe anxiety than the students whose self-assessment of academic pressure was very low. This suggested that students with high self-assessment of academic pressure were at greater risk of moderate or severe anxiety. A study in Shandong Province of China showed that rural students and female self-assessment of academic pressure were higher. As a result, they were at higher risk of moderate or severe anxiety [52]. Reducing students’ academic pressure was suggested to prevent students at high risk from developing moderate or severe anxiety. Parents and teachers need to pay attention to students and identify students with high-level academic stress early, and prompt interventions that will likely prevent or ameliorate anxiety [51].

Some limitations of the present research should be considered when interpreting its findings. First of all, cross-sectional data limits the interpretation of the findings as causal claims cannot be made. Where possible, future research should aim for longitudinal data collection to better understand the process of anxiety development in childhood and adolescence. Second, anxiety and school-related factors were assessed using self-report surveys. Third, only adolescents attending school were included. Future research should aim to obtain data from different sources (such as teacher reports or investigator reports) to corroborate the findings. 

Notwithstanding, the current study was unique in its person-centered approach to studying anxiety and its school-related factors correlate in a sample of junior high school students. LPA revealed three distinct anxiety groups, including mild anxiety, moderate anxiety, severe anxiety [28,53]. Females are more likely to be moderate or severe anxiety than males. Students with high self-assessment of academic pressure were at greater risk of moderate or severe anxiety. Students were at lower risk for severe anxiety when school mental health work was adequate.

## 5. Conclusions

The overall level of anxiety among rural students is high. The survey revealed that gender differences in anxiety rates, finding that girls were more anxious than boys. Higher levels of anxiety were observed among students from female students, students with more excellent academic performance, and students with higher academic pressure. Lower levels of anxiety were observed among students whose school mental health work was adequate. We have provided insight into which groups are particularly vulnerable to anxiety. Our findings may enable parents, teachers, and schools to develop more specific and efficient strategies for handling the anxiety of adolescents. Ultimately, our findings may help China’s leaders better target investments and create policies aimed at improving the mental health of students in rural China.

## Figures and Tables

**Table 1:** Parameters of fit for 1–5 profile solutions for anxiety of junior high school students in the rural areas of Jiangxi Province.

The Number of Profiles	AIC	BIC	ABIC	Entropy	LMRT*_P_*	VLMR*_P_*	BLRT*_P_*	Sample < 5%
1	33,703.032	33,779.800	33,729.057	——	——	——	——	No
2	32,141.132	32,261.192	32,181.796	0.802	<0.001	<0.001	<0.001	No
3	31,572.277	31,735.558	31,627.580	0.816	<0.001	<0.001	<0.001	No
4	31,431.690	31,638.193	31,501.632	0.773	0.049	0.047	<0.001	No
5	31,340.553	31,590.277	31,425.134	0.749	0.187	0.183	<0.001	No

AIC: Akaike information criterion, BIC: Bayesian information criterion, ABIC: same-size adjusted Bayesian information criterion, LMRT*_P_*: the *p*-value of the Lo–Mendell–Rubin likelihood ratio test, VLMR*_P_*: the *p*-value of the Vuong–Lo–Mendell–Rubin likelihood ratio test, BLRT*_P_*: the *p*-value of bootstrapped likelihood ratio test.

**Table 2:** Demographic characteristics and anxiety category of junior high school students in the rural areas of Jiangxi Province.

Variable	Respondents	Mild Anxiety	Moderate Anxiety	Severe Anxiety	*χ^2^*	*p*
Gender					31.337	<0.001
Male	467 (51.89)	110 (63.58)	275 (54.56)	82 (36.77)		
Female	433 (48.11)	63 (36.42)	229 (45.44)	141 (63.23)		
Age (year)					5.990	0.648
12	126 (14.00)	21 (12.14)	72 (14.29)	33 (14.80)		
13	157 (17.44)	26 (15.03)	93 (18.45)	38 (17.04)		
14	258 (28.67)	55 (31.79)	145 (28.77)	58 (26.01)		
15	179 (19.89)	40 (23.12)	98 (19.44)	41 (18.39)		
16	180 (20.00)	31 (17.92)	96 (19.05)	53 (23.77)		
Left-behind children (LBC)					0.088	0.957
Yes	263 (29.22)	49 (28.32)	148 (29.37)	66 (29.60)		
No	637 (70.78)	124 (71.68)	356 (70.63)	157 (70.40)		
Health education course					3.531	0.171
Yes	453 (50.33)	95 (54.91)	240 (47.62)	118 (52.91)		
No	447 (49.67)	78 (45.09)	264 (52.38)	105 (47.09)		
School mental health work is adequate					21.938	<0.001
Yes	387 (43.00)	84 (48.55)	231 (45.83)	72 (32.29)		
No	112 (12.44)	15 (8.67)	53 (10.52)	44 (19.73)		
Not sure	401 (44.56)	74 (42.77)	220 (43.65)	107 (47.98)		
Self-assessment of academic performance					33.794	<0.001
Excellent	41 (4.56)	15 (8.67)	13 (2.58)	13 (5.83)		
Good	398 (44.22)	84 (48.55)	229 (45.44)	85 (38.12)		
Pass	301 (33.44)	52 (30.06)	185 (36.71)	64 (28.70)		
Fail	160 (17.78)	22 (12.72)	77 (15.28)	61 (27.35)		
Self-assessment of academic pressure					86.854	<0.001
Very high	141 (15.67)	13 (7.51)	66 (13.10)	62 (27.80)		
High	236 (26.22)	27 (15.61)	137 (27.18)	72 (32.29)		
General	433 (48.11)	101 (58.38)	258 (51.19)	74 (33.18)		
Low	41 (4.56)	8 (4.62)	27 (5.36)	6 (2.69)		
Very low	49 (5.44)	24 (13.87)	16 (3.17)	9 (4.04)		
Total	900 (100.00)	173 (100.00)	504 (100.00)	223 (100.00)		

**Table 3:** The variable assignment summary for logistic regression analysis.

Factors	Variable Name	Factor Assignment
Dependent variables		
Anxiety	Y_1_, Y_2_	Mild anxiety (reference): Y_1_ = 0, Y_2_ = 0
		Moderate anxiety: Y_1_ = 1, Y_2_ = 0
		Severe anxiety: Y_1_ = 0, Y_2_ = 1
Independent variables		
Gender	X_1_	0 = Female (reference); 1 = Male
Age (year)	X_2_, X_3_, X_4_, X_5_	12 (reference): X_2_ = 0, X_3_ = 0, X_4_ = 0, X_5_ =0
		13: X_2_ = 1, X_3_ = 0, X_4_ = 0, X_5_ =0
		14: X_2_ = 0, X_3_ = 1, X_4_ = 0, X_5_ =00
		15: X_2_ = 0, X_3_ = 0, X_4_ = 1, X_5_ =0
		16: X_2_ = 0, X_3_ = 0, X_4_ = 0, X_5_ =1
Left-behind children (LBC)	X_6_	0 = No (reference); 1 = Yes
Health education classes	X_7_	0 = No (reference); 1 = Yes
School mental health work is adequate	X_8_, X_9_	Not sure (reference): X_8_ = 0, X_9_ = 0
		Yes: X_8_ = 1, X_9_ = 0
		No: X_8_ = 0, X_9_ = 1
Self-assessment of academic performance	X_10_, X_11_, X_12_	Fail (reference): X_10_ = 0, X_11_ = 0, X_12_ = 0
		Excellent: X_10_ = 1, X_11_ = 0, X_12_ = 0
		Good: X_10_ = 0, X_11_ = 1, X_12_ = 0
		Pass: X_10_ = 0, X_11_ = 0, X_12_ = 1
Self-assessment of academic pressure	X_13_, X_14_, X_15_, X_16_	Very low (reference): X_13_ = 0, X_14_ = 0, X_15_ = 0, X_16_ = 0
		Very high: X_13_ = 1, X_14_ = 0, X_15_ = 0, X_16_ = 0
		High: X_13_ = 0, X_14_ = 1, X_15_ = 0, X_16_ = 0
		General: X_13_ = 0, X_14_ = 0, X_15_ = 1, X_16_ = 0
		Low: X_13_ = 0, X_14_ = 0, X_15_ = 0, X_16_ = 1

**Table 4:** The multifactor analysis of anxiety of junior high school students in the rural areas of Jiangxi Province by logistic regression.

Variable	Moderate Anxiety		Severe Anxiety	
	OR	OR 95%*C.I.*	OR	OR 95%*C.I.*
Gender		Female as Ref.		Female as Ref.
male	0.649 *	(0.444,0.949)	0.262^*^	(0.166,0.415)
Age(year)		12 as Ref.		12 as Ref.
13	0.750	(0.385,1.459)	0.733	(0.339,1.587)
14	0.587	(0.310,1.110)	0.495	(0.231,1.061)
15	0.647	(0.354,1.183)	0.461 *	(0.224,0.950)
16	0.975	(0.493,1.928)	0.818	(0.365,1.834)
Left-behind children (LBC)		No as Ref.		No as Ref.
Yes	0.966	(0.646,1.445)	0.884	(0.546,1.434)
Health education course		No as Ref.		No as Ref.
Yes	0.752	(0.503,1.125)	1.187	(0.734,1.920)
School mental health work is adequate		Not sure as Ref.		Not sure as Ref.
Yes	1.047	(0.695,1.577)	0.562 *	(0.341,0.926)
No	1.130	(0.587,2.174)	1.898	(0.928,3.883)
Self-assessment of academic performance		Fail as Ref.		Fail as Ref.
Excellent	0.377 *	(0.143,0.989)	0.431	(0.148,1.255)
Good	0.763	(0.432,1.349)	0.362 *	(0.192,0.683)
Pass	0.989	(0.548,1.784)	0.460 *	(0.237,0.892)
Self-assessment of academic pressure		Very low as Ref.		Very low as Ref.
Very high	6.523 *	(2.666,15.957)	11.579 *	(4.164,32.194)
High	6.122 *	(2.773,13.515)	5.894 *	(2.280,15.233)
General	3.037 *	(1.492,6.181)	1.573	(0.650,3.811)
Low	3.555 *	(1.243,10.166)	1.143	(0.285,4.579)

* *p <* 0.05.

## Supplementary Materials

Supplementary File 1
