# Supplementary material for: A Latent Profile Analysis of Anxiety among Junior High School Students in Less Developed Rural Regions of China
Source: Int J Environ Res Public Health. 2020 Jun 8;17(11):4079. doi: 10.3390/ijerph17114079 (PMC7312008; doi:10.3390/ijerph17114079)
Supplement: Supplementary File 1 [file ijerph-17-04079-s001.pdf]

南昌大学第二附属医院医学研究
伦理委员会证明

袁兆康 申报的课题（ 留守儿童健康策略调查 ）经我院伦理委员会审查，研究内容符合伦理原则，同意申报该项目，如获资金。伦理委员会将按照国家相关法规进行监督。

南昌大学第二附属医院
医学研究伦理委员会
2003年5月20日
